# Is Crime Decreasing?

**Published:** 1896-03-14

**Authors:** 


### Is Crime Decreasing?

We are often inclined to congratulate ourselves on the decrease in the number of criminals tried at our assizes and immured in our prisons, but we do not often stop to inquire if a smaller number of criminals means a less amount of crime. The diminution of trials may mean indeed an improvement of public morality, but it may imply no more than a greater leniency on the part of prosecutors. The decrease of the number of prisoners may show only the increasing moderation of judges and the leniency of modern codes. An alteration in the law may make a vast diminution in the number of convictions at any time. When hanging for sheep-stealing was abolished, the number of executions was bound to grow less.

The development of criminology as a science has had a great effect in producing this apparent decrease in crime. The public conscience, realising that in many cases the criminal is only in part responsible for his deeds, often refuses to convict him. How many magistrates, feeling that drunkenness is a disease rather than a crime, dismiss the drunkard with an admonition which they know to be futile, rather than condemn him to a short imprisonment, which they know will do no more than restore his health for his next bout? The general feeling is against short periods of incarceration. If you condemn the criminal, especially the occasional criminal who sins through a comprehensible temptation and not from mere anti-social instinct, let the punishment be a detention long enough to destroy his criminal associations and to teach him some honest craft. Some good may result; but mere imprisonment, while it may protect society for the time from his ravages, does nothing for the criminal himself.

It is the occasional criminal who is the hope and pride of prisoners' aid societies and the Howard Association. "There, but for the grace of God, goes John Wesley," said the great preacher, as he saw a murderer going to be hanged, and the most virtuous man is conscious of occasional impulses within himself which, given temptation and opportunity, would lead him to the criminal's dock. Therefore, good antecedents and a previously blameless life are always held up as extenuating circumstances in a criminal. In one sense they are the reverse, for good influences should have kept him from crime; but the underlying sentiment is that here is a man not irredeemably bad, a man who will avail himself of another chance, if it be given him to retrieve the errors of the past. The genuine criminal is irretrievably bad. He is bad from his birth, born and not made so, a product of pre-natal forces as much as a responsible being. He is not insane, as tried by the tests of the ordinary alienist, yet he is destitute of the prudence, the justice, the industry of the average man. He will steal or starve, but he will not work; he will kill a fellow creature to gain a prize that would hardly tempt a child, or to avenge an injury for which one would not kick a dog. The moral sense is lacking in him, and without making too much of anthropometry it cannot be denied that these measurements show the criminal to be different in certain well-defined forms from the majority of his fellows. Professors Lombroso and Ferri agree that "the relapsing criminal is the rule rather than the exception." The latter reckons that about 60 per cent. of the prisoners whose cases he investigated had relapsed after a first conviction. The relapses are more frequent among the minor offences, such as petty theft, drunkenness, and vagrancy, but they are not unknown even in cases of murder, and tend to show that criminality is a condition rather than a special act.

Crime, therefore, will not decrease through either leniency or severity of the law while criminals endure, although changes in the code, the increasing charity of judges, founded on scientific grounds, or the tenderness of juries founded on sentimental ones, may lessen the number of convictions. Of course, heroic remedies may bring down the number, as, it is said, the wholesale hanging of beggars by the Tudors lessened vagrancy for generations to come. But, short of killing the criminal, is there any way of eradicating crime?

Once a criminal always a criminal; if that be so, the death penalty might be inflicted for every offence. A criminal yourself, your children will be criminals; if that be so, you should be prevented from being a parent. These solutions of the problem may be practical, but they are such as the conscience of civilised and Christian man cannot entertain. What then remains?

It seems not unreasonable, when a man has proved by repeating an offence once or twice that he is a confirmed criminal, to condemn him to perpetual imprisonment, even though his sins be not of the most heinous kind. For not only the drunkard, but the thief might there be an asylum prepared, not as for a sinner but as for an invalid. The aim of society is not to punish the criminal, and it cannot cure him; but it protects itself by putting him out of harm's way. The vindictive idea is banishing from our penal codes, but the protective one is not fully established, and meanwhile a vague and capricious leniency too often obtains.

But the progress of the world is itself working unconsciously to save the world from many crimes. Highway robbery has almost ceased, not because highwaymen have seen the error of their ways, but because the swiftness of railway travelling makes the crime difficult. Occasional murders in a railway carriage there are, and these could be almost wholly ended by the universal adoption of Pullman cars. It is true that in the land of Pullman a train is occasionally "held up" by freebooters, but only in those parts where population is sparse and the rate of trains slow. The westward march of civilisation will soon end all that. Similarly, better systems of book-keeping have largely checked, if they have not cured, embezzlement, and the unjust steward would find his tampering with accounts less easy to-day than in the days of our Lord. The photograph and the telegraph make it continually more difficult for the thief to be long successful in his career, and the discoveries of chemistry lay endless traps in the path of even the cleverest poisoner. The development of knowledge everywhere embarrasses the path of the criminal, and to this we must look for the diminution of crime in the greatest degree; from preventive societies and institutions for bringing up children in honest paths we may hope for some good results, but from either excessive severity or excessive leniency of penal laws we must expect next to nothing.

